# Patient Portals as a Tool for Health Care Engagement: A Mixed-Method Study of Older Adults With Varying Levels of Health Literacy and Prior Patient Portal Use

**DOI:** 10.2196/jmir.7099

**Published:** 2017-03-30

**Authors:** Taya Irizarry, Jocelyn Shoemake, Marci Lee Nilsen, Sara Czaja, Scott Beach, Annette DeVito Dabbs

**Affiliations:** ^1^ School of Nursing University of Pittsburgh Pittsbrugh, PA United States; ^2^ Department of Psychology Ohio State University Columbus, OH United States; ^3^ University Center for Social and Urban Research University of Pittsburgh Pittsburgh, PA United States

**Keywords:** patient portals, patient participation, telemedicine, aged, health literacy, access to information, patient preference

## Abstract

**Background:**

Growing evidence that patient engagement improves health outcomes and reduces health care costs has fueled health providers’ focus on patient portals as the primary access point for personal health information and patient-provider communication. Whereas much attention has been given to identifying characteristics of older adults who do and do not adopt patient portals and necessary adaptions to portal design, little is known about their attitudes and perceptions regarding patient portal use as a tool for engagement in their health care within the context of health literacy, experience navigating Web-based health information, and previous patient portal use.

**Objective:**

The specific aims of this study were to explore attitudes toward portal adoption and its perceived usefulness as a tool for health care engagement among adults (65 years and older) who have varying levels of health literacy and degrees of prior patient portal use.

**Methods:**

A phone survey of 100 community dwelling adults gathered sociodemographic, health, and technology related information. Older adults were purposefully selected for 4 follow-up focus groups based on survey responses to health literacy and previous patient portal use. A mixed-method approach was used to integrate phone survey data with thematic analysis of 4 focus groups. Due to variability in attitudes between focus group participants, an individual case analysis was performed and thematic patterns were used as the basis for subgroup formation.

**Results:**

Differences in health literacy, comfort navigating health information on the Web, and previous portal experience explained some but not all differences related to the 7 themes that emerged in the focus groups analysis. Individual cases who shared attitudes were arranged into 5 subgroups from least to most able and willing to engage in health care via a patient portal. The subgroups’ overall portal adoption attitudes were: (1) Don’t want to feel pushed into anything, (2) Will only adopt if required, (3) Somebody needs to help me, (4) See general convenience of the portal for simple tasks and medical history, but prefer human contact for questions, and (5) Appreciates current features and excited about new possibilities **.**

**Conclusions:**

Most of the older adults are interested in using a patient portal regardless of health literacy level, previous patient portal adoption, or experience navigating health information on the Web. Research targeting informal caregivers of older adults who are unable or unwilling to engage with information technology in health care on their own is warranted. Health care organizations should consider tailored strategies to meet the needs of older adults (and their informal caregivers) and explore alternative workflows that integrate patient portal information into phone conversations and face-to-face contact with health care providers.

## Introduction

Growing evidence that patient engagement improves health outcomes and reduces health care costs [1] coupled with government reforms to promote efficiency, quality, and safety [2] has fueled health providers’ focus on patient portals as the primary access point for personal health information and patient-provider communication [3]. Additionally, health systems have added convenience features such as prescriptions refills and appointment scheduling. Consequently, consumer adoption of patient portals is becoming increasingly critical for receipt of quality health care including interactions with health providers outside of clinical visits and quick access to one’s personal health information.

Although the intention of patient portals is to promote patient engagement, numerous large-scale survey studies have demonstrated that older adults are less likely to adopt portals even though they utilize the greatest proportion of health care resources [4-6]. Low adoption rates are most pronounced among older adults who have less access to and experience with technology, less education, and who demonstrate low health literacy and numeracy skills [7,8] **.** These barriers have been aptly described as the “gray digital divide” [9].

Qualitative studies examining the known barriers that contribute to the gray digital divide discuss a strong need for supplementary support to assist vulnerable patients with portal navigation, particularly those with limited health literacy [10,11]. In addition, user-satisfaction, usability, and task analysis studies [12-15] have focused on older adults’ experiences navigating portal functionality and evaluating their performance of specific tasks. Results from these studies indicate that previous computer experience and adequate health literacy and numeracy are strong contributing factors to ones’ ability to successfully perform health management tasks using a patient portal.

Whereas much attention has been given to identifying the characteristics of older adults who do and do not adopt patient portals and necessary adaptions to portal functionality, little is known about their attitudes and perceptions regarding portal use as a tool for engagement in their health care within the context of health literacy level, experience navigating Web-based health information, and previous patient portal use. A better understanding of the relationships connecting these concepts could help health care systems align their organizational practices and system design (ie, people, process, and technology) [16] to better meet the unique needs of the older adult populations they serve [8,10]. Therefore, the specific aims of this study were to explore attitudes toward portal adoption and its perceived usefulness as a tool for health care engagement among older adults with varying levels of health literacy and degrees of prior patient portal use.

## Methods

### Design

**Figure 1:**
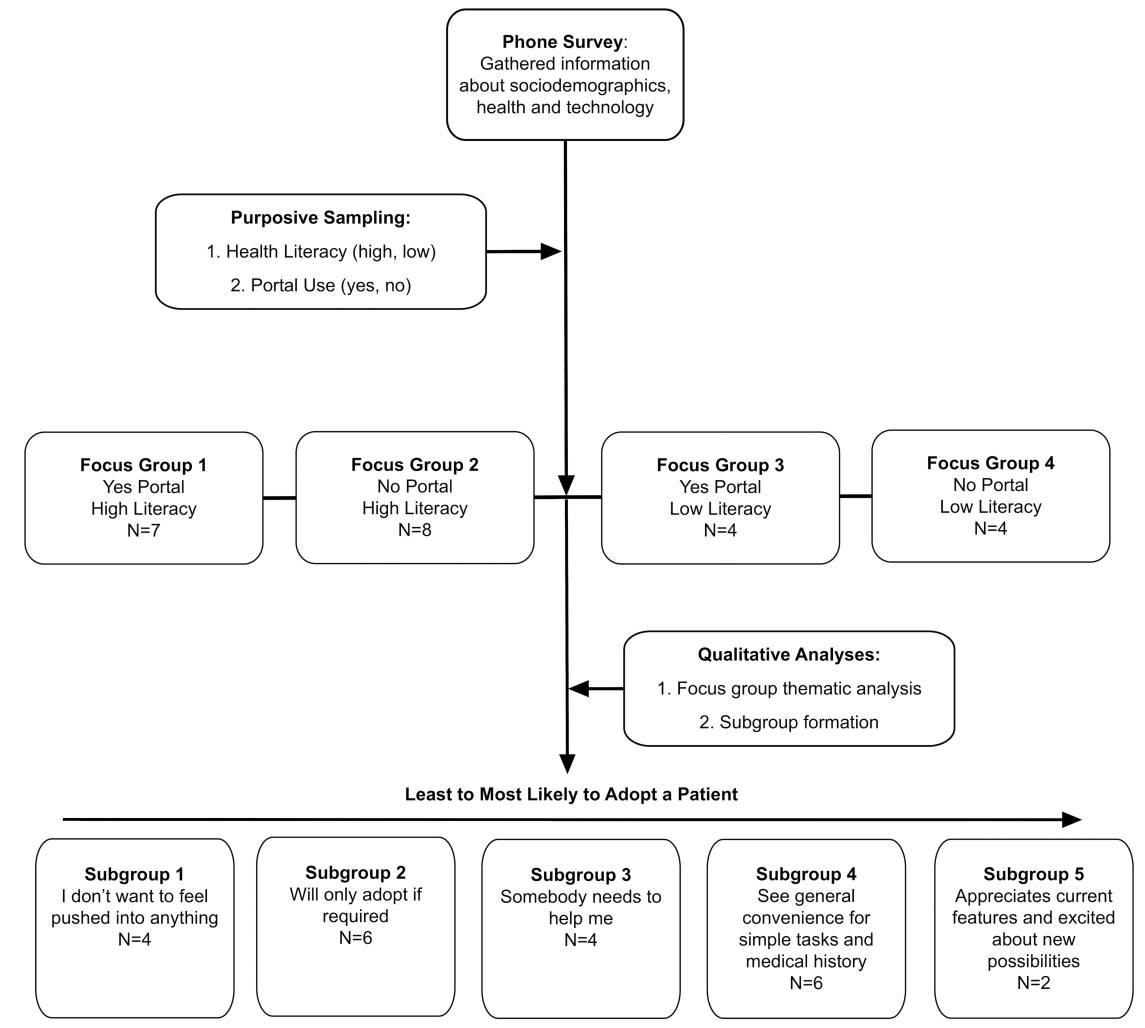
Overview of mixed-method study design.

A mixed-method study design was chosen for the purpose of complementarity [17], meaning findings from quantitative and qualitative methods were integrated in a complementary fashion to produce a more complete understanding of the phenomena of interest. Integration occurred at multiple stages. First, a phone survey was used to assess sociodemographic and other health- and technology-related characteristics in a sample of 100 community dwelling older adults. Second, subsamples of older adults were purposefully selected to participate in follow-up focus groups based on responses to health literacy score and patient portal use. Third, the quantitative data from the phone survey and thematic analysis results of the focus group were integrated to form a rich description of older adults’ experiences with navigating health information on the Web and attitudes toward the patient portal’s usefulness as a tool for engagement in their health care. Finally, distinct patterns among individuals across all 4 focus groups were identified, resulting in the formation of subgroups ranging from least to mostly likely to adopt a patient portal. Refer to Figure 1 for an overview of the study design. Ethics approval for this research was obtained through the University of Pittsburgh Institutional Review Board (REN16060086/PRO15050313).

### Sample

A convenience sample of English-speaking community-dwelling older adults (defined as living in an independent residence, ie, not in an assisted living facility or nursing home) who were at least 65 years of age and cognitively equipped to answer a full battery of questions were recruited from the institutional review board (IRB)–approved research registry maintained by University of Pittsburgh’s University Center for Social and Urban Research (UCSUR). The registry (n=1000) was assembled from a variety of population-based surveys conducted by UCSUR in the Pittsburgh area; thus, it is generally representative of the regional population.

A final sample of 100 registry participants was contacted by phone by experienced interviewers and a 45-minute survey was completed. Each participant received US $10 for participation. Seventy-five of the phone survey participants agreed to be contacted for possible participation in a follow-up focus group. Those who agreed were then stratified by level of health literacy and portal use based on responses to two survey items: (1) whether or not the participant had experience using a patient portal (yes, no), and (2) level of health literacy (high, low). Purposive sampling was used to include representation of participants in terms of age, race, and gender in each of the 4 focus groups. The first 10 participants who met criteria for one of the 4 groups and were willing and able to commit to a common meeting date and time were invited to participate.

### Phone Survey Data Collection

The selection of survey measures was based on evidence-based criteria related to the gray digital divide. Instruments were selected from the core battery for demographics, health and disability from the Quality of Life Technology (NSF 0540865), and the core battery from the Center for Research and Education in Aging and Technology Enhancement (CREATE) which includes sociobehavioral factors proposed by the Institute of Medicine for inclusion in the electronic health record [18].

#### Sociodemographics

Demographic variables included age (years), gender, race (white, African American, other), marital status (single or divorced or widowed, married or living with partner), education (high school degree or general educational development [GED], associate or vocational training, bachelor’s, master’s degree), and income (less than or US $14,999, US $15,000-US $39,000, US $40,000-US $69,000, US $70,000 or more).

#### Health Status and Disease Burden

Health characteristics included the number of comorbidities endorsed from a list of 12 potential conditions with the option of “other” and responses to 2 questions about perceived health status: (1) “In general, would you say your health is” (poor or fair, good, very good, or excellent), and (2) “How often are your daily activities limited due to your physical health” (never or seldom, sometimes, often, or always).

#### Health Literacy

The brief health literacy screen (BHLS), a subjective health literacy screening measure [19], includes 3 Likert-scale questions addressing one’s confidence completing medical forms, reading hospital materials, and understanding written health information. On the basis of previous research [20,21], the response cutoff that optimized sensitivity and specificity for low health literacy was ≤ 3 (ie, “somewhat” or “some of the time”), meaning only participants who scored a 4 or 5 on all 3 questions were considered to have adequate health literacy (although we refer to the dichotomy as high and low literacy for simplicity in the study results). These questions have been previously shown to correlate with the Short Test of Functional Health Literacy in Adults (S-TOFHLA) and Rapid Estimate of Adult Literacy in Medicine as criterion standards [20-22].

#### Level of Engagement in Health and Health Care

The short form Patient Activation Measure (PAM-13) created by Hibbard and colleagues, a reliable and valid tool that is widely used in both research and clinical settings [23-25], was used to measure engagement. The PAM-13 includes 13 items with 4 response options ranging from 1(disagree strongly) to 4 (agree strongly). The raw score is then converted to an overall score ranging from 0-100 using the table provided in PAM licensing materials [25]. Scores are categorized into one of four levels of activation ranging from least to most active: level one (47.0 or lower), patients believe taking an active role in their health is important but are unprepared for this role; level two (47.1 to 55.1), patients have some knowledge but still struggle to manage their medical conditions; level three (55.2 to 67.0), patients begin to take action in terms of self-management but do not have the skills to support or sustain their behavior; and level four (67.1 or above), patients have adopted self-management behaviors and work on maintaining them in stressful life situations.

#### Technology Use and Attitudes

Two items of the Technology and Computer/Web Experience Questionnaire [18] were included in the survey. First, use of technology was measured by whether participants ever searched on the Web for health-related information: “During the past three months, did you or someone who helps you look online for information about any of the following topics: health condition or treatment, medications, health service availability, health professionals, and or health care facilities?” (yes or no). Second, they were asked to rate their attitudes about technology on a scale from 1 (not at all) to 10 (completely) for each of the following descriptors: “To what extent do you believe that technology makes life:” (1) easy and convenient, (2) complicated, (3) gives people control over their daily lives, (4) makes people dependent, (5) comfortable, (6) stressful, (7) brings people together, (8) makes people isolated, (9) increases personal safety and security, and (10) reduces privacy. Negative descriptors were reverse scored and a final score was an average of the total sum, thus a higher score means a more positive attitude.

#### Portal Use

Previous use of a patient portal was determined by a yes or no response to the question, “Have you ever used a patient portal on your own?” Answers were dichotomized as no or yes (all or some of the time). Further clarification was gained by asking, “Does someone help you access the portal or access the portal on your behalf?” If yes, some or all the time **.**

### Focus Group Data Collection

#### Health Literacy

Since previous research had identified health literacy and numeracy as contributing factors for patient engagement and patient portal use [14,26], in addition to screening for health literacy using BHLS, we administered the newest vital sign (NVS) to focus group participants. The NVS is a 6-item objective health literacy measure of both reading comprehension and numeracy abilities [27]. Each participant was taken to a private space before the focus group discussions and given a copy of the nutrition label and the NVS questions. Scoring of the NVS is based upon number of correct answers with 0-1=high likelihood of limited health literacy, 2-3=possibility of limited health literacy, and 4-6=adequate health literacy skills. The NVS time to completion was also recorded; any participant taking longer than 7 minutes to complete the screening questions is considered to have low health literacy. The NVS has been previously shown to correlate with the Short Test of Functional Health Literacy in Adults (S-TOFHLA) and Rapid Estimate of Adult Literacy in Medicine short form (REALM-SF) as criterion standards [27,28].

#### Attitudes Toward Technology and Portal Use

Experienced moderators facilitated the focus groups using a topical guide geared toward understanding participants’ attitudes toward technology, experiences of accessing health-related information on the Web, and perceived ability to use and benefit from patient portal functions. In addition to the open discussion, an interactive patient portal demonstration, developed by Czaja and colleagues at CREATE [12,29], University of Miami, was used to stimulate conversation about common features of patient portals and to seek participants’ reactions.

Each of the 4 focus group sessions lasted approximately 1 hour and participants were provided light refreshments and US $40 for participation. The focus group discussions were audio recorded, transcribed verbatim by trained transcriptionists, and reviewed for accuracy by the focus group facilitators before being uploaded in to Atlas.ti version 7.5 (Scientific Software Development GmbH), the software program chosen to organize data for coding and visualization. The transcripts were supplemented with session notes and linked to participant survey data.

### Phone Survey Analysis

Descriptive statistical analyses of all variables of interest (demographics, health characteristics, engagement, health literacy, technology use and attitudes, and portal use) were organized by level of study participation: (1) participants who only completed the phone survey (n=26), (2) those contacted for possible follow-up focus group participation (n=51), and (3) focus group participants (n=23). In order to determine differences between the 3 groups, chi-square test of association was used for dichotomous variables (gender, race, marital status, technology use for health-related activities, health literacy, and portal use). Post hoc testing using standardized adjusted residuals was used to determine the source of any significant result using a threshold of ±2 [30]. Kruskal-Wallis H was used for ordinal and continuous variables (age, education, income, all health characteristics, engagement and technology attitudes). Subsequent pairwise comparisons were performed using Dunn [31] procedure with a Bonferroni correction for multiple comparisons and adjusted *P* values were presented. SPSS version 24.0 software (SPSS Inc) was used for all analyses.

### Focus Groups Analysis

The thematic analysis [32] of qualitative transcript data of the focus groups was initiated by a lead coder who used open coding to describe the views of participants regarding the following topics: (1) experience with technology for health-related information and (2) impressions about the patient portal demonstration and its potential usefulness in promoting personal engagement in health care. A second coder then reviewed the initial codes and added new codes when she felt existing codes were needed. Both coders met to reach consensus on final codes. A third coder joined the team to collapse codes into themes through a process of consensus. Descriptive statistical analyses of demographics, health characteristics, engagement, health literacy, technology use and attitudes, and portal use were performed to in order to identify quantitative differences and similarities between focus groups.

### Case Analysis

The stratification of focus groups by patient portal use and health literacy explained some but not all differences in attitudes toward accessing Web-based health information, portal adoption, and perceptions of usefulness of patient portal functionality. Due to the variability in attitudes between individuals within the focus groups, an individual case analysis was performed by all 3 coders in order to identify potential patterns. The case analysis began by linking passages from the transcript to individuals, which made it possible to connect the codes from individuals to the themes identified in the focus groups. Individuals’ thematic patterns were then displayed in a matrix and subgroups were identified based upon the similarities and differences between them (Table 5). In addition, characteristics including demographics, health characteristics, health literacy, and technology use and attitudes were calculated for each subgroup in order to identify possible quantitative similarities and differences.

## Results

### Quantitative Results by Level of Study Participation

Survey results by level of study participation and group difference statistics are displayed in Table 1. Significant group differences were found for race (*P*=.03), searching on the Web for health information (*P*=.01), education (*P*=.01), income (*P*=.001), health status (*P*=.003), and engagement (*P*=.001). No statistically significant group differences were found for any of the other variables (age, gender, marital status, limited due to physical health, health literacy, or attitude toward technology). The post hoc analysis revealed statistically significant differences between the phone survey group, the follow-up call, and focus group attendees, but not between the follow-up call group and focus group attendees (see Tables 2 and 3). Post hoc testing indicated more African American or other focus group attendees than would be expected by chance (adjusted residual 2.6). This finding supports the success of the purposive sampling technique, which was meant to encourage the most racially diverse participant representation possible. Post hoc testing also indicated fewer survey participants searched on the Web for health information than would be expected by chance (adjusted residual −2.6). The purposive sampling method did not control for technology-related variables, therefore, this finding suggests that people who are more familiar with accessing Web-based health information may be more willing to participate in research related to technology in health care.

**Table 1:** Between group differences by level of study participation: phone survey, follow-up call, focus group.

Participant characteristics				Phone survey (n=26)	Follow-up call (n=51)	Focus group (n=23)	Group differences
Sociodemographics							
**Age in years (n=100), Mean (range)**				76.58 (65-93)	74.69 (65-97)	72.61 (65-82)	X^2^_2_=2.6, *P*=.27
**Gender (n=100), n (%)**	Female			12 (46.2)	30 (58.8)	13 (52.2)	X^2^_2_=0.8, *P*=.79
**Race (n=99), n (%)**							X^2^_2_= 6.9, *P*=.03
	White			21 (80.8)	44 (86.3)	13 (56.5)	
	African American			2 (7.7)	7 (13.7)	6 (26.1)	
	Other			3 (11.5)	0 (0)	3 (13.0)	
	Refused^a^					1 (4.3)	
**Marital status (n=100), n (%)**	Married or living with partner			8 (30.8)	28 (54.9)	12 (52.2)	X^2^_2_=4.2, *P*=.12
**Education (n=99), n (%)**							X^2^_2_=8.9, *P*=.01
	Less than high school degree			2 (7.7)	2 (3.9)	0 (0)	
	High school degree or general educational development			12 (46.2)	12 (23.5)	3 (13.0)	
	Associate or vocational training			6(23.1)	14 (27.5)	9 (39.1)	
	Bachelor’s degree			3 (11.5)	12 (23.5)	8 (34.8)	
	Master’s degree			2 (7.7)	11 (21.6)	3 (13.0)	
**Income in US $ (n=96), n (%)**							X^2^_2_=13.0, *P*=.001
	<$14,999			7 (26.9)	6 (11.8)	3 (13.0)	
	$15,000-$39,999			14 (53.8)	13 (25.5)	4 (17.4)	
	$40,000-$69,999			5 (19.2)	17 (33.3)	8 (34.8)	
	>$70,000			0 (0)	13 (25.5)	4 (17.4)	
	Refused^a^			2 (50)	2 (3.9)		
Health characteristics							
**Current health status (n=100), n (%)**							X^2^_2_=11.3, *P*=.003
	Poor to fair			16 (61.5)	10 (19.6)	5 (21.7)	
	Good			6 (23.1)	26 (51.0)	9 (39.1)	
	Very good to excellent			2 (7.69)	15 (29.4)	9 (39.1)	
**Daily activities limited due to physical health (n=100), n (%)**							X^2^_2_=4.4, *P*=.11
	Never			5 (19.2)	14 (27.5)	12 (52.2)	
	Seldom			5 (19.2)	12 (23.5)	13 (13.0)	
	Sometimes			14 (53.8)	19 (37.3)	6 (26.1)	
	Often to always			2 (7.7)	6 (11.8)	2 (8.7)	
Number of comorbidities (n=100), mean (range)				2 (0-5)	1.94 (0-6)	1.56 (0-5)	X^2^_2_=2.4, *P*=.08
**Health literacy (n=100), n (%)**							
	Brief health literacy screen (Hi)			9 (34.6)	28 (54.9)	15 (65.2)	X^2^_2_=4.9, *P*=.08
Engagement							
**Patient Activation Measure (PAM, n=100; 0-100) mean (range)**				59.49 (41.70-80.0)	71.71 (37.30-100.0)	77.05 (52.90-100.0)	X^2^_2_ 15.1, *P*=.001
	PAM level 1, n (%)			3 (11.5)	1 (2.0)	0 (0)	
	PAM level 2, n (%)			4 (15.4)	4 (7.8)	1 (4.3)	
	PAM level 3, n (%)			13 (50)	19 (37.3)	6 (26.1)	
	PAM level 4, n (%)			6 (23.1)	27 (52.9)	16 (69.6)	
**Technology attitude (n=99)**							
	Searched on the Web for health-related information (yes), n (%)			9 (34.6)	35 (68.6)	12 (52.2)	X^2^_2_=8.9, *P*=.01
	Technology attitudes (score 0-10), mean (range)			5.72 (2.7-9.6)	6.33 (3.4-8.8)	6.26 (4-8.5)	
**Portal use (n=100), n (%)**							
	Ever use a patient portal on your own (yes)			0 (0)	25 (49.0)	11 (47.8)	X^2^_2_=19.8, *P*=<.001
	**Someone helps you use portal^b^**						
		Yes, all of the time		0 (0)	3 (5.9)	0 (0)	
		Yes, sometimes		6 (23.1)	4 (7.8)	6 (26.1)	
	**Someone accesses the portal on your behalf^b^**						
			Yes, all of the time	2 (7.7)	4 (7.8)	0 (0)	
			Yes, sometimes	5 (19.2)	3 (5.9)	4 (17.4)	

^a^Participants chose not to supply information.

^b^Not analyzed due to sample size.

**Table 2:** Post hoc analysis of dichotomous variables.

Dichotomous Demographic Variables^a^		Phone survey only	Follow-up call	Focus group attendee
**Race**				
	White	21 (0.3)	44 (1.9)	13 (−2.6)
	African American or Other	5 (−0.3)	9 (−1.9)	7 (2.6)
**Searched on the Web for health information**				
	Yes	9 (−2.6)	35 (2.7)	12 (−0.5)
	No	17 (2.6)	15 (−2.7)	11 (0.5)

^a^Adjusted residuals appear in parenthesis next to observed frequencies.

**Table 3:** Post hoc analysis of ordinal variables.

Ordinal Demographic Variables	Adjusted significance, *P* value	Group^a^ (mean rank)
Education	.03	PS (35.84), AFC (53.74)
	.02	PS (35.84), FGA (57.33)
	>.99	AFC, FGA
Income	.001	PS (31.54), AFC (54.03)
	.02	PS (31.54), FGA (52.50)
	>.99	AFC, FGA
Current health status	.007	PS (35.15), AFC (55.09)
	.01	PS (35.15), FGA (57.67)
	>.99	AFC, FGA
Engagement (patient activation measure)	.007	PS (32.69), AFC (53.87)
	.001	PS (32.69), FGA (63.15)
	.59	AFC, FGA

^a^PS: phone survey, AFC: follow-up call, FGA: focus group attendee.

### Quantitative Results by Focus Group

Of the 40 volunteers who committed to one of the 4 focus groups, only 23 attended the sessions. There was no follow-up with the participants who didn’t make it to the sessions, so the reason for not attending in unknown. The yes portal, low literacy group and no portal, low literacy group had 4 participants, the yes portal, high literacy group had 7, and the no portal, high literacy group had 8. Survey results per focus group are displayed in Table 4. The no portal, low literacy group had the widest age range (65-82 years) and included 2 participants in their early eighties. Three out of 4 of the focus groups had an equal proportion of males and females. The no portal, high literacy group had fewer males (n=3) and was the most racially diverse with 3 African Americans and 3 others who did not identify themselves as white, but did not specify. The majority of participants in all focus groups had education beyond high school or GED, with half having a college degree. Both yes portal groups had more white and married participants, as well as more participants with higher average income.

The majority of participants reported their perceived health status was “good,” to “excellent,” and only 2 in the yes portal high literacy group and 2 in the no portal low literacy group reported “poor or fair” health. All the groups had at least one participant report his or her physical condition limits daily activities “sometimes.” The no portal low literacy group had 1 participant who reported “often” being physically limited. This same participant reported having 5 comorbidities, where the range across all other groups was 0-3.

The NVS scores for the high literacy groups (yes portal and no portal) ranged from 2-6 and 0-6, respectively. The NVS scores for the low literacy groups (yes portal and no portal) ranged from 5-6 and 0-5, respectively. The BHLS and NVS scores were not always in alignment, meaning fewer participants were found to have high literacy using the NVS than with the BHLS screening instrument. No notable differences were found in PAM scores, searching on the Web for health-related information, or attitudes toward technology with the exception that for the technology items the average scores for the no portal low literacy group were slightly lower.

**Table 4:** Characteristics of the sample by focus group.

Patient characteristics		Group 1 Yes portal, high literacy (n=7)	Group 2 No portal, high literacy (n=8)	Group 3 Yes portal, low literacy (n=4)	Group 4 No portal, low literacy (n=4)
Sociodemographics					
Age in years, mean (range)		73.29 (66-80)	73.88 (66-80)	69.00 (66-73)	72.50 (65-82)
**Gender, n (%)**					
	Male	4 (57.1)	3 (37.5)	2 (50)	2 (50)
**Race, n (%)**					
	White	5 (71.4)	2 (25)	3 (75)	3 (75)
	African American	2 (28.6)	3 (37.5%)	0 (0)	1 (25)
	Other	0 (0)	3 (37.5)	1 (25)	0 (0)
	Refused^a^		1(12.5)		
**Marital status, n (%)**					
	Single or divorced or widowed	2 (28.6)	5 (87.5)	0 (0)	2 (50)
	Married or living with partner	5 (71.4)	1 (12.5)	4 (100)	2 (50)
**Education, n (%)**					
	High school degree or general educational development	1 (14.3)	1 (12.5)	0 (0)	1 (25)
	Associate or vocational training	2 (28.6)	4 (50)	0 (0)	1 (25)
	Bachelor’s degree	2 (28.6)	3 (37.5)	2 (50)	1 (25)
	Master’s degree	2 (28.6)	0 (0)	2 (50)	1 (25)
**Income in US $, n (%)**					
	<$14,999	1 (14.3)	2 (25)	0 (0)	0 (0)
	$15,000-$39,999	1 (14.3)	2 (25)	1 (25)	0 (0)
	$40,000-$69,999	1 (14.3)	3 (37.5)	1 (25)	3 (75)
	>$70,000	2 (28.6)	0 (0)	2 (50)	0 (0)
	Refused^a^		1 (12.5)		1 (25)
Health characteristics					
**Current health status, n (%)**					
	Poor to fair	2 (28.6)	1 (12.5)	0 (0)	2 (50)
	Good	3 (42.9)	3 (37.5)	3 (75)	0 (0)
	Very good to excellent	2 (28.6)	4 (50)	1 (25)	2 (50)
**Daily activities limited due to physical health, n (%)**					
	Never	4 (57.1)	5 (62.5)	1 (25)	2 (50)
	Seldom	1 (14.3)	1 (12.5)	0 (0)	1(25)
	Sometimes	1 (14.3)	2 (25)	3 (75)	0 (0)
	Often to always	1 (14.3)	0 (0)	0 (0)	1(25)
Number of comorbidities mode (range)		1 (1-2)	1 (0-5)	1 (1-2)	2 (0-2)
Health literacy					
Newest vital sign (possible score 0-6) Mean (range)^b^		4.71 (2-6)	3.2 (0-6)	5.5 (5-6)	2.75 (0-5)
Newest vital sign time to complete (minutes, seconds) Mean (range)^b^		3.28 (3.44- 5.38)	5.43 (2.7- 16.33)	3.46 (2.53-7.0)	5.12 (2.41-7.23)
Engagement					
**PAM^c^** **(possible score 0-100)** **Mean (range)**		77.80 (56.40-100.00)	82.74 (52.90-100.00)	66.40 (56.40-77.50)	76.21 (56.40-100.00)
	PAM^c^ level 1, n (%)	0 (0)	0 (0)	0 (0)	0 (0)
	PAM level 2, n (%)	0 (0)	1 (12.5)	0 (0)	0 (0)
	PAM level 3, n (%)	2 (28.6)	1 (12.5)	2 (50)	1 (25)
	PAM level 4, n (%)	5 (71.4)	6 (75)	2 (50)	3 (75)
**Technology attitude**					
	Searched on the Web for health-related information (yes)	4 (57)	4 (50)	2 (50)	2 (50)
	Technology attitudes (possible score 0-10), mean (range)	6.60 (5-8.5)	6.45 (5-8)	6.68 (5-8)	4.88 (4-6)
**Portal Use**					
	Someone helps you use portal some or all of the time (yes), n (%)	3 (42.9)	1 (12.5)	1 (25)	1 (25)
	Someone accesses the portal on your behalf some or all of the time (yes), n (%)	1 (14.3)	1 (12.5)	1 (25)	1 (25)

^a^Participants chose not to supply information.

^b^The newest vital sign (NVS) measured during the focus group sessions only.

^c^PAM: patient activation measure.

### Qualitative Results by Focus Group

Seven major themes were identified and arranged in order from least to most positive experience and attitudes toward technology use for health care and portal adoption. The 7 themes included: (1) limited or poor relationship with technology, (2) fears and frustrations with technology and portal, (3) prefers phone over secure messaging for communication (outside of clinical visit), (4) willing to adopt the portal with support, (5) good relationship with technology, (6) Internet as source of health information, and (7) portal is helpful. The following detailed explanation of each of the 7 themes includes all the original codes that informed the theme and is framed by the focus group stratification criteria (previous portal use and health literacy level).

#### Limited or Poor Relationship With Technology

Participants in every focus group expressed at least some degree of negativity toward technology use in health care as well as in everyday encounters. Difference in reasons why varied by literacy level. The most common negative sentiment among participants in the high health literacy groups was difficulty troubleshooting without having access to live technical support and feeling pressured to adopt new communication methods (eg, instant messaging, video calls) that seemed unnecessary. In contrast, many participants in the no portal, low health literacy group mentioned having had little experience using computers and did not have the Internet access in their homes. Many no portal, low literacy group participants noted that they had no computer training as part of their job and retired before computers were a regular part of the working environment. Many felt afraid of making a mistake or felt stigmatized by their lack of knowledge and therefore either avoided using computers all together or relied heavily on family members to help them.

#### Fear and Frustration With Technology and Portal

Fears about personal health data security risks were shared in every focus group. Those participants in the high health literacy groups were more articulate in describing specific instances in which they felt uncomfortable, whereas those in the low health literacy groups spoke more generally about security fears. Despite the level of specific risk described, some participants cited the risk as being too great, whereas others felt it was an everyday risk we all must “get used to.” In all instances, participants felt that speaking over the phone with a trusted health care professional was the safest and most secure way to share health information.

Participants with more experience navigating the health care system and seeing several medical specialists shared experiences where they felt burdened by the task of circulating the most current health information even though all health care providers had access to the same electronic health record. These people also described instances where medical history in the patient portal was outdated or incorrect which led to a sense of frustration, as it was not clear what action should be taken to correct it. A few participants from the high literacy group who had experience using the patient portal secure message function recounted instances in which they either did not get a response, or they were not entirely certain that they understood the response. In either case, they experienced anxiety and frustration. They also expressed discomfort with the idea that other staff aside from the primary health care provider could respond to personal secure messages via the patient portal because they did not personally know them.

#### Prefers Phone Over Secure Messaging for Communication (Outside of Clinical Visit)

Those who experienced anxiety and frustration using the patient portal’s secure message feature were convinced that phone was the only way to communicate, ask questions, and clarify information. The no portal high literacy group voiced the most opposition to secure messaging in health care encounters and the most strongly in support of direct clinician communication by phone. When probed further, there were instances where the participant felt comfortable speaking with a nurse, nurse practitioner, or physician assistant (as opposed to an office manager) as long as they worked closely with his or her provider and felt they had enough clinical expertise. Those with no portal experience in both the high and low literacy groups were more positive about the potential for secure messaging and the idea of having access to their provider outside the clinical visit.

#### Willing to Adopt Portal With Support

Participants in both the low health literacy groups expressed an interest in portal training. Those with less experience with computers had less confidence in their ability to learn and expressed an interest in having someone access the system on their behalf. The high literacy portal users expressed fear and anxiety around doing something wrong and had recommendations for a “task specific” training in which users would learn the purpose of each function (eg, information searches, secure messaging, appointment, and medication refill requests) and gain hands-on experience with navigation, use, and troubleshooting.

#### Happy to Engage With Technology

Participants who were eager to learn new things, had exposure to computer use at work, or sought out resources to learn computer skills, expressed appreciation for the conveniences of technology. They were eager to share what they had learned in terms of accessing computers outside of the home, where to take computer classes, and ways to troubleshoot. The level of enthusiasm and interest did not parallel levels of health literacy or portal use. However, participants in the high literacy no portal group were the most philosophical in their debate about technology degrading the quality of human relationships, and were the least willing to discuss potential benefits to their personal lives. High literacy portal users who had negative experiences with information sharing among providers or negative experiences with the portal expressed both positive and negative feelings about their relationship with technology in the health care setting, yet generally felt the positives outweighed the negatives.

#### Internet as a Good Source of Health Information

The degree to which participants felt the information they received from their providers was enough for them was variable across all focus groups. Some participants from the high literacy portal user group felt the portal lacked individualized information and that it was more helpful to search on the Web. In contrast, some low literacy groups (portal and nonportal users) believed the health information presented in the portal was the more trusted and reliable, and therefore it made them feel more confident about the information in comparison to looking up information on the Internet.

#### Sees Portal as Beneficial

Convenience features of the portal were perceived as useful by participants regardless of whether they had used a particular function before and whether or not they could personally see themselves using it. Overall, participants in good health thought it was helpful to have all their personal medical information, including clinician contact numbers, all in one place for easy reference, but felt that other more complex functions would be helpful for people who were managing a serious illness or chronic condition or were acting as a caregiver. In most cases, they considered their current non-technological methods (eg, paper copy of their post-visit summary) to be satisfactory. Participants who had experience with serious illness and chronic disease management were vocal about the convenience of having timely access to lab results and having them stored electronically, but felt that the kind of communication they needed to have with their providers was too involved for secure messaging.

### Qualitative Case Results

Individual cases who shared attitudes toward engaging in one’s health care via technology and portal adoption were arranged into 5 subgroups from least to most able and willing to engage in health care via a patient portal. All individual participants were placed in a subgroup except for 1 from the no portal, high literacy group who could not be categorized due to minimal verbal participation, and therefore scarcity of codes attributed to her. Table 5 presents the matrix of individuals in relationship to the themes and demonstrates the prevalence of themes in each focus group. Table 6 presents a description of the 5 subgroups, including the number of individuals from each of the 4 original focus groups, and summarizes the general attitudes toward adoption of technology for health care engagement and adoption of the portal.

**Table 5:** Endorsement of themes by individual participants within focus group.

Focus group^a^	Individual participant	Limited or poor relation-ship with technology	Fears and frustrations with technology (may extend to portal)	Prefers phone as primary mode of communication	Portal willing with support	Good relationship without technology	Internet as source of health information and education	Portal is helpful
No portal low literacy	Carol	X	X	X				X^b^
	Henry	X	X	X				X^c^
	Sheila			X		X	X	X
	Willy	X	X	X				X^c^
No portal high literacy	Elsie	X	X	X	X			X^c^
	Mick		X	X	X			X^c^
	Brian			X				X^c^
	Gerald	X		X				
	May			X				X^c^
	Francis	X						
	Jane		X		X			X^c^
	Mary							X^c^
Yes portal low literacy	John		X	X		X	X	X
	Lynn					X		X
	Rick	X	X	X				X^c^
	Terry		X	X				X^c^
Yes portal high literacy	Rob	X	X	X				X^c^
	Sue		X	X		X	X	X
	Gary		X	X				X^c^
	Tim	X		X	X			
	Anne							X
	Ray		X	X	X	X	X	X
	Lily	X						X^c^

^a^Themes in columns left to right from most negative to most positive.

^b^Considered the portal to be generally helpful but not for their personal use.

^c^Considered the portal to be helpful only for viewing lab results.

**Table 6:** Attitudes of subgroups arranged according to the least (subgroup 1) to most likely (subgroup 5) to adopt health technologies and patient portal.

Subgroup	Original focus group					Attitudes toward adoption of technology for health care engagement		Attitudes toward adoption of the portal
	YPHL^a^	YPLL^b^	NPHL^c^	NPLL^d^				
Subgroup 1 n=4	2			2	Don’t think the benefits are worth the hassle or risk		I don’t want to feel pushed into anything	
Subgroup 2 n=6		1	5		Satisfied as things are		Will only adopt if required	
Subgroup 3 n=4	2		1	1	Technology is the way of the future, but too difficult to learn new things		Somebody needs to help me	
Subgroup 4 n=6	2	2	1	1	Comfortable with technology, but prefer to talk to a person for personal health-related issues		Sees general convenience of the portal for simple tasks and medical history	
Subgroup 5 n=2	1	1			Thrilled with technology for information and communication with no reservations.		Appreciates current features and excited about new possibilities	

^a^YPHL=yes portal, high literacy.

^b^YPLL=yes portal, low literacy.

^c^NPHL=no portal, high literacy.

^d^NPLL=no portal, low literacy.

### Quantitative Case Results

The smallest of the 5 subgroups (n=2) comprised women who expressed complete comfort with technology and were previous portal users. Although neither of them had a college degree, they both scored a 6 out 6 on the NVS and completed the literacy measure in less than 3 minutes (far less time than of any other subgroup where at least half of the group had a college degree). They were both married, nearly the same age (70 and 71 years), reported an income over US $70,000 a year, and expressed having good, but not excellent health. All other subgroups were more diverse in terms of demographics, NVS scores, and attitudes toward technology. Subgroup 3 expressed interest in using technology yet acknowledged they needed assistance to do so. This group had the widest range in age, NVS scores and completion time, and the highest number of comorbidities.

## Discussion

### Principal Findings

This study used a mixed-method approach to gain greater insight into the experiences and attitudes toward the patient portal as an engagement tool among older adults with varying levels of health literacy and patient portal experience. Numerous topics revealed in the focus groups were consistent with current literature on the adoption of patient portals by older adults including fears about security issues, interest in convenience features (eg, appointment scheduling, prescription refills), and lack of access to a computer or Internet as a major barrier to portal adoption [33]. The unique contributions of this study are the findings regarding attitudes of older adults toward adoption of a patient portal for engagement in health care, specifically in the context of health literacy and previous experience navigating Web-based health information.

Focus group participants were assembled based on health literacy level (assessed using the BHLS) and previous experience using a patient portal. This stratification explained some but not all differences in attitudes toward portal adoption and perceptions of usefulness of patient portal functionality. Neither the health literacy scores from the BHLS nor the NVS was directly correlated with previous patient portal adoption or perceived usefulness except in the case of subgroup 5 which comprised 2 women who scored 6 out of 6 on the NVS, expressed complete comfort with technology, and were regular portal users. The qualitative findings revealed that health literacy was a contributing factor to confidence accessing and evaluating health care information on the Web (this evidence is corroborated by Diviani [34]). However, health literacy was not directly related to one’s motivation to engage in health care via a patient portal as evidenced by the case analysis and subsequent subgroupings.

Qualitative findings also revealed that previous experience accessing and evaluating health care information on the Web was not entirely correlated with prior patient portal adoption or perceptions about its potential usefulness. In fact, some members of the no portal focus groups (both high and low health literacy) who did not have experience navigating health information on the Web were motivated to explore their health care institution’s patient portal following the patient portal simulation, that included examples of how the patient portal functionality could be helpful within a personally relevant context. This finding is supported by Melenhorst, Rogers, and Bouwhuis [35] who identified the “decisive role of perceived benefits” as the main motivator of adoption among older adult users.

Presently, most patient portals are introduced via an email with a time-sensitive link and sign-on instructions, or a postcard given in a clinical visit or sent through the mail. These methods may be appropriate for younger generations who are more comfortable with navigating the Web-based environment including sharing personal information and social interaction. However, this research demonstrates that older adults require an initial introduction that highlights contextually relevant benefits and addresses their particular needs and concerns. Both high and low health literacy groups felt that specific task-based training was an important, yet lacking, resource that would help build confidence and understanding of when, why, and how to navigate the features included in patient portals.

Additionally, deliberate outreach and tailored training of informal caregiver proxy users is recommended in the case of older adults who recognize and appreciate the potential benefits of the patient portal as a tool for engagement, yet lack computer access or perceive themselves as unfit to manage their own health information. The potential role of informal caregivers as a key factor to improve access and use of patient portals by older adults who are unable to engage on their own is recognized in the literature [35-37], yet very little research exists regarding the experience of acting as a proxy-user, effective strategies for encouraging portal adoption among proxy-users, and what design features could be enhanced to encourage engagement with both the older adult of concern and his or her health care provider [38].

Whereas a patient portal adoption campaign tailored to older adults could convince and encourage greater numbers of older adults to use patient portals, many participants in this as well as other studies [7,39] express fear that they won’t always understand the personal health information available to them and felt that the secure message function was a poor substitute for direct clinician-patient interaction necessary to clarify things. This sentiment remained strong across all focus groups as evidenced by the case analysis matrix (Table 5) and was especially true for older adults managing complex conditions. Frequent portal users also described instances where medical history in the patient portal was outdated or incorrect which led to a sense of frustration and concern, as it was not clear what action to take to correct it. A possible solution for these issues, concerns, and frustrations may be restructuring care team workflows. Examples include making it possible for patients and informal caregivers to call and talk to a clinic representative who can answer simple questions, verify and update information presented in the patient portal (which would then update the electronic health record), and triage more serious issues when appropriate. In addition, further integration of patient portal use during face-to-face encounters with health care providers and phone interactions with clinic representatives could transform the patient portal into an information resource for all parties and may incentivize older adults and caregivers to use it as a tool for health care engagement outside of clinical visits. Refer to Table 7 for a brief description of findings, implications, and recommendations.

### Reliability, Validity, Trustworthiness, and Rigor

Quantitative and qualitative procedures were performed according to the assumptions of each paradigm. Survey methods, including sampling, use of validated measures, the collection of data using standard formatting by trained phone interviewers, and standard analysis techniques ensured reliability and validity of quantitative analysis [40]. Trustworthiness and rigor of qualitative focus group phase of the study included purposive sampling to ensure as much heterogeneity with regard to participant characteristics as possible [41]. The focus groups were led by an experienced facilitator using a semistructured guide and field observer. All focus group sessions were audiorecorded, transcribed verbatim, and open coding was initially performed to provide thick description of exchanges during the focus groups [42]. Three researchers performed thematic analyses independently and interpretation of the findings and meaning making was achieved by consensus [43]. Quantitative and qualitative data sources were integrated using matrices [44] and merged according to recommended methods [40].

**Table 7:** Summary of findings, implications, and recommendations.

Findings	Implications	Recommendations
Health literacy: A contributing factor to confidence accessing and evaluating health care information on the Web. Yet, not directly related to one’s motivation to engage in health care via a patient portal.	Health literacy is not a primary barrier to patient portal adoption, but may impact confidence in navigating its features.	Offer specific task-based training to build confidence and understanding of when, why, and how to navigate the features included in patient portals.
Perceptions of portal usefulness: Not entirely correlated with prior patient portal adoption or previous experience accessing and evaluating health care information on the Web.	Older adults are motivated to adopt a portal when the initial introduction highlights contextually relevant benefits and addresses their particular needs and concerns.	Create a patient portal adoption campaign tailored to the needs and concerns of older adults.
Some willing adopters are unable: Some older adults appreciate the potential benefits of the patient portal as a tool for engagement, yet lack computer access or perceive themselves as unfit to manage their own health information.	The potential role of informal caregivers as a key factor to improve access and use of patient portals by older adults who are unable to engage on their own is supported here and recognized in the literature.	Design deliberate outreach and tailored training of informal caregiver proxy users.
Portal as source of information, but not a stand-alone solution: Many feared they wouldn’t always understand portal information and felt secure messaging was a poor substitute for direct clinician-patient interaction necessary to clarify things.	Most of the older adults believe the portal is convenient for simple tasks and medical history, but is not sufficient as a stand-alone engagement tool.	Explore alternative workflows that integrate portal use into face-to-face clinical encounters and offer access to personnel with the skills to review and respond to questions over the phone or triage more serious issues if appropriate.
Errors in portal information are a source of concern: Frequent users found outdated or incorrect medical history in the portal and were unsure what to do about it.	The usefulness of the portal is diminished when the information is not accurate and promotes dissatisfaction when no clear avenue of correction is available.	Explore alternative workflows that offer access to personnel with ability to change, up-date, and validate missing or inaccurate portal information.

### Limitations

This study used a convenience sample of older adults from a Pittsburgh regional research registry. Whereas a strong effort was made to achieve the most representative sample of those in jeopardy of experiencing the gray digital divide, the comparison between phone survey, follow-up, and focus group attendees revealed statistically significant differences between survey participates and focus group participants. Most notably, focus group participants were more educated, with higher income, better health status, higher engagement scores, and more experience with searching for health information on the Web than survey participants. Alternative recruitment methods designed to target older adults that are least likely to be interested in focus group participation, such as semistructured interviews over the phone or home visits, are warranted. However, the sample characteristics of the focus group participants are representative of the average older American adult population, and therefore helpful for understanding general attitudes toward technology in health care and portal adoption.

In regards to the focus group sample, characteristics of interest were generally comparable across groups; however, both low literacy groups were nearly half the size of the high literacy groups despite having had the same number of participant invitations accepted. A more equal representation of low literacy participants may have added further clarification of the differences in attitudes and preferences as compared with the high literacy groups. Also, 3 of the 4 groups were 75% white. Having more input from a wider range of racial backgrounds may have added more detail regarding differences in adoption attitudes toward the patient portal related to race. Additionally, this study was conducted in English, which limited our findings to fluent English speakers. Whereas most patient portals are currently in English only, continued work in other languages is necessary to meet the needs of our increasingly diverse communities. Descriptive statistics of the focus groups and subgroups were explored and reported in order to provide a rich multidimensional description; however, the small sample size of the focus groups and subgroups did not allow for statistical inferences between participant characteristics and the attitudes toward health care engagement via a patient portal.

Finally, the question, “Does someone help you access the portal or access the portal on your behalf?” (If yes, some or all of the time) was asked in the survey phase; however due to the minimal number of positive answers within any of the groups, it was not possible to explore the concept in a mixed-method approach (although the data is reported). Instead, the role of the caregiver proxy was discussed in detail qualitatively. Further research focused on addressing older adult informal caregivers’ attitudes and experiences as proxy users is needed.

### Conclusions

The study used quantitative and qualitative methods in a complementary fashion to produce a more complete understanding of older adults’ attitudes toward using a patient portal as a health care engagement tool within the context of health literacy, previous experience with patient portal, and accessing Web-based information. Only a minority of older adults believe that the security risks or trouble learning something new is not worth it; most of the older adults are interested in using a patient portal regardless of health literacy level, previous patient portal adoption, or experience navigating health information on the Web. Health care organizations should consider the following strategies to align people, process, and technology in order to meet the needs of the older adults they serve: (1) create a patient portal adoption campaign tailored to the needs of older adult so that the benefits are communicated in a contextually relevant way, (2) offer task-specific training so they feel they have the support they need to confidently use the functionality, (3) specifically target informal caregiver proxy users as part of the adoption campaign and training, and (4) explore alternative workflows that give patients access to personnel with the skills to review and respond to questions over the phone about personal health information within the portal, as well as change, up-date, validate missing or inaccurate information, and triage more serious issues when appropriate. Such organizational strategies would transform the patient portal from a repository of information with a secure message function to a tool designed to support engagement, information sharing, and enhanced communication between care teams, patients, and informal caregivers.

## Abbreviations

BHLS: brief health literacy screen
GED: general educational development
NVS: newest vital sign
PAM-13: Patient Activation Measure
REALM-SF: Rapid Estimate of Adult Literacy in Medicine short form
S-TOFHLA: Short Test of Functional Health Literacy in Adults
